# Vaccination Causes Consumption

**Published:** 1898-11

**Authors:** Wm. B. Clarke

**Affiliations:** Indianapolis, Ind.


### VACCINATION CAUSES CONSUMPTION.

Wm. B. Clarke, M. D., Indianapolis, Ind.

The *Medical Century* considers it an "absurd claim" to intimate that vaccination causes consumption. As I advanced the "absurd" claim in The Homœpathiic Physician, it is now in order to adduce authority for the statement.

It is certain that scrofulous and tuberculous diseases have increased since the introduction of cow-pox, and that the vaccine virus favors particularly the prevalence of various forms of scrofula.—*Copland's Medical Dictionary*.

That tubercle or scrofula can be engendered or intensified by vaccination is no new theory. Dr. Squirrel suggested the idea some seventy years ago, and the experiments of Cohnheim, Fox, and Toussaint confirm his hypothesis. That the danger is a real one seems almost affirmed as a scientific fact. —*Winterburn's Vaccination*, page 136 (experiments and statistics afterward cited).

Consumption follows in the footsteps of vaccination as directly as an effect ever follows a cause. The vaccine poison being the product of decaying animal tissue and often tuberculous in character, must naturally produce its like wherever it finds the suitable opportunity.—*Dr. Alexander Wilder in The Metaphysical Magazine*, May, 1898.

*The Medical Times and Gazette*, London, as long ago as January 1st, 1854, called attention to the fact that consumption had widely spread since the introduction of vaccination.

Vaccination is a poisoning of the blood.—*Dr. Constantine Hering*, 1878.

Dr. Nittinger, of Stuttgart, testified before the Royal Vaccination Commission to the widespread causation of disease, including phthisis, by vaccination.

Dr. Charles Creighton cites twelve cases of consumption that within his knowledge were caused by vaccination. As he wrote the article "Pathology" in the *Encyclopedia Britannica*, he may be presumed to know a hawk from a hernshaw.

Dr. Perron, of the French Legion of Honor, in an article on vaccination in the army, in the *Gazette Hebdomodaire des Sciences Medicales*, in 1890, says: "In all European armies vaccination is the order of the day. On arrival with the corps the young soldiers are re-vaccinated. Now the military statistics of all countries show an enormous proportion of tuberculosis, especially during the first and second year after enlistment. Tuberculosis shows itself in the garrisons of all countries with frequency before which one might well despair. Whence come these attacks, so sudden, so numerous, upon subjects that but a few months before were rightly declared fit for military service? We believe we must seek the reason in the vaccination of recruits. If we examine the events of the last century or so we can show a constant increase of tuberculosis, a fact never hitherto satisfactorily explained. Now, in spite of all the incessant progress in public and private hygiene, and material improvements, it tends more and more to rise to the rank of pestilence. If tuberculosis, in spite of all sanitary precautions, has multiplied its attacks during the last hundred years, it is, we submit, because vaccination has come to create for it a propitious soil."

And if the editor of the *Medical Century* must have something more severely "official," I append a resolution adopted at the International Congress of Hygiene and Demography, held at Madrid Easter week, as taken from the *Medical Record:*

"Inasmuch as tuberculosis is easily transmitted by vaccination when it is done directly from the calf, the International Council of Hygiene and Demography asks that in all nations represented at the meeting, the practice should be adopted of using in official vaccination stations only the lymph of calves which have been examined *post mortem* and pronounced to be free from tuberculosis."

Of course this would be rough on the calves and disastrous to our beef supply—nor would it avoid the danger if adopted.

Dr. Fisher may think it "absurd" to claim that vaccination *may* predispose the race toward cancer, but fails to enlighten us as to what it is that has quadrupled the deaths from that disease in the United States and England in the last forty years, and almost doubled them in New York in the last ten, these being official figures. On page 117 of the September, 1898, *Medical Advance*, Dr. Wickens, in an article on medicine in England, states that "the deaths from cancer in England have increased 113 per cent. in the last thirty years."

Many additional testimonies regarding this "absurd claim"

could easily be cited did space permit. Nor have I time just now to devote to the real absurdities indulged in concerning the article criticized.

